# Occupational violence against nursing staff in the surgical wards of Murzuq locality hospitals, Libya (2024)

**DOI:** 10.1186/s12912-025-02870-y

**Published:** 2025-02-27

**Authors:** Eltagi Elsadeg Sulliman Rahama, Mohammed Elmadani, Malak Mokhtar Mohammed, Zainab Ali Osman Eqrer

**Affiliations:** 1 Faculty of Nursing, Fezzan University, Fezzan, Libya; 2 Department of Surgical Nursing, Faculty of Nursing Sciences, University of El Imam El Mahdi, Kosti, Sudan; 3 Department of Epidemiology, Faculty of Public Health, University of El Imam El Mahdi, Kosti, Sudan; 4 Jamhuriya Research Center, Jamhuriya University of Science and Technology, Mogadishu, Somalia; 5 Doctoral School of Health Sciences, Faculty of Health Sciences, University of Pecs, Pecs, Hungary; 6 Murzuq Hospital, Murzuq, Libya

**Keywords:** Health personnel, Job satisfaction, Libya, Nurses, Surgical nursing, Workplace violence

## Abstract

**Background:**

Occupational violence is a significant issue for nurses worldwide, impacting their well-being, job satisfaction, and patient care quality. This study investigated the prevalence, types, and effects of such violence on nursing staff in surgical wards in Murzuq locality hospitals, Libya, to inform effective prevention strategies.

**Methods:**

This cross-sectional study examined occupational violence against nursing staff in surgical wards across three hospitals in Murzuq, Libya. Using a convenience sampling approach, data were collected from 50 nurses in general surgical and emergency surgical wards over four weeks through a self-administered questionnaire. The variables included demographic data (gender, experience) and information on violence types, sources, impacts, and reduction strategies. The data were analysed via SPSS v27, with descriptive statistics applied. For inferential statistics, the chi-square test and Fisher’s exact test were conducted at a significance level of 0.05. Ethical approval was obtained from the academic research committee of Fezzan University, and informed consent was secured from all participants.

**Results:**

Verbal insults (60%) and discrimination (64%) were the most reported types of violence. Patient companions (68%) were identified as the primary source, followed by patients (40%) and colleagues (28%). Occupational violence significantly impacted nurses’ lives, with 62% reporting stress, 48% experiencing decreased job satisfaction, and 28% considering leaving their jobs. Additionally, 40% reported difficulty concentrating due to violence. Significant associations were found between gender and sources of violence (*p* ≤ 0.001) and between years of experience and types, sources, and impacts of violence (*p* ≤ 0.001).

**Conclusion:**

These findings underscore the need for gender-sensitive interventions, comprehensive training, and support mechanisms to address workplace violence. Future research should explore the long-term effects of violence on nursing staff and the effectiveness of tailored interventions in different healthcare settings. The results highlight the critical role of improving work conditions and organizational policies to enhance workplace safety for nurses.

## Background

Occupational violence in healthcare, particularly in surgical wards, is a global issue that threatens the safety and well-being of healthcare providers [1]. Defined as any abuse, threat, or assault against staff during work, occupational violence encompasses physical attacks, verbal abuse, and psychological threats [1, 2]. The World Health Organization (WHO) reported that 8–38% of healthcare workers experience physical violence, with even higher rates of psychological abuse [3, 4]. Nurses are disproportionately affected among healthcare workers because of their close and sustained interactions with patients, especially in high-stress environments such as surgical wards [5–7]. Surgical wards present unique risks for occupational violence due to their high-pressure environment and the vulnerability of patients recovering from surgery. Factors such as severe pain, emotional distress, and adverse reactions to anaesthesia frequently contribute to violent incidents [8–10]. Studies have demonstrated that surgical wards are among the healthcare units with high rates of violence against nurses [11–15].

Occupational violence affects nurses in both high- and low-income countries worldwide. For example, 66% of nurses in Australia reported experiencing workplace violence within a year [16], whereas 50% of nurses in Ghana and over 50% in China reported similar experiences, including widespread verbal abuse [17, 18]. In the United States, more than 20% of nurses face physical assault annually, with emergency departments and surgical wards being frequent hotspots [19]. However, incidents of violence are often underreported due to fears of retaliation, guilt, or the perception that violence is an unavoidable part of the job [20]. Factors at the individual, management, and organizational levels influence the underreporting of workplace violence (WPV). Nurses fear consequences such as blame or retaliation for reporting incidents [20, 21]. Additionally, a lack of knowledge about reporting processes or insufficient training further discourages reporting [20]. Unsupportive management cultures, including a lack of visible response or consequences following reports, contribute to nurses’ reluctance to report incidents [20, 22]. The absence of penalties for perpetrators also fosters the perception that reporting is ineffective [23]. Many healthcare organizations fail to implement clear policies and training on WPVs, contributing to confusion and further reluctance to report [20]. Inadequate or cumbersome reporting systems deter nurses from reporting incidents [20].

In African countries, workplace violence (WPV) against nurses is particularly alarming, with prevalence rates ranging from 9 to 100% [24]. For example, in Arabic-speaking African countries, 62.3% of nurses reported experiencing violence, including verbal abuse (51.2%), threats (23.3%), bullying (22.9%), physical violence (15.1%), and sexual harassment (10.3%) [25]. Factors contributing to WPV include weak hospital security, nurse shortages, and increased workload [5, 26]. Predictors such as workplace characteristics and nurses’ prior experiences highlight the multifaceted nature of the problem [25]. Despite its prevalence, workplace violence remains underreported due to fear of blame, inadequate support systems, and ineffective reporting mechanisms [21]. The consequences are far-reaching and include psychological distress, burnout, and reduced job performance, compromising patient safety and exacerbating global nursing shortages [27–29].

In addition to external violence, lateral violence and hostility among colleagues present a significant challenge in healthcare settings [30, 31]. Lateral violence in nursing, including bullying, verbal abuse, and psychological harassment, creates a hostile work environment and negatively impacts patient care [32]. It is linked to cultural misunderstandings, lack of leadership intervention, and insufficient organizational justice. At Jinnah Hospital Lahore, 51.9% of nurses reported rarely experiencing respect from coworkers, with cultural factors contributing to workplace bullying [33]. In the U.S., bullying among nurses is widespread, affecting staff well-being and patient safety [34]. Its consequences include lower productivity, increased absenteeism, and financial losses due to high turnover [35]. Organizational justice plays a key role in reducing lateral violence, yet many nurse leaders lack awareness and training to address it effectively [31, 36–39]. Evidence-based interventions, such as leadership training and improved communication, are essential for prevention [34]. Providing nurse managers with training in fair management strategies and implementing regulations can help combat workplace violence [36].

Programmes such as the Surgical Ambassador Program in Australia demonstrate the potential of comprehensive strategies to reduce occupational violence and its impacts [10, 40]. Effective approaches include staff training, policy reforms, and fostering organizational cultures that encourage reporting and accountability [41–43]. Legislative efforts, such as the Workplace Violence Prevention for Health Care and Social Service Workers Act in the U.S., underscore the importance of institutional commitment to healthcare worker safety [40, 42].

While the literature extensively documents the prevalence and impacts of occupational violence in healthcare, particularly among nurses, there is a notable gap in understanding the specific dynamics and preventive strategies in high-stress environments such as surgical wards. Studies often focus on broader healthcare settings or single forms of violence, leaving a limited understanding of how systemic factors, workplace culture, and staff training intersect to exacerbate violence in surgical wards. Moreover, research addressing the dual challenges of underreporting and lateral violence remains fragmented.

This study aims to bridge these gaps by providing a comprehensive analysis of occupational violence in surgical wards, focusing on its multifaceted causes, consequences, and preventive measures. By integrating perspectives from diverse regions and highlighting both external and lateral violence, this research seeks to offer actionable insights for policy development, institutional reforms, and staff training. The findings will contribute to filling critical knowledge gaps and advancing practical solutions to protect healthcare workers and improve patient care outcomes.

## Methods

### Study design

This study employed an explorative, cross-sectional design to investigate occupational violence against nursing staff working in surgical wards through a questionnaire-based approach. A cross-sectional design was chosen for this study because it allows for efficient data collection at a single point in time, making it ideal for assessing the prevalence and characteristics of occupational violence within the context of surgical wards at Murzuq Locality Hospitals. This design enabled the identification of key factors associated with workplace violence without the need for long-term tracking. Moreover, the study aimed to assess the general occurrence and impact of violence rather than compare different groups. Given the exploratory nature of the research and practical constraints, the cross-sectional design was considered the most suitable approach for addressing the study’s objectives.

### Study area

This study was conducted in Murzuq locality, situated in the Fezzan region of southwest Libya. The study targeted nurses working in three selected hospitals, with a total of 194 nursing staff. Murzuq General Hospital employed 128 nurses, Tasawa Rural Hospital had 26 nurses, and Aqar Otba Hospital had 40 nurses. The hospitals varied in size and capacity: Murzuq General Hospital had 120 beds, whereas Tasawa and Aqar Otba Hospitals, which primarily serve as rural healthcare facilities, each had 10 beds [44]. The selection of hospitals was based on convenience, considering proximity to the researchers’ living areas and ease of accessibility. This decision was necessary due to logistical constraints, including time, resource availability, and feasibility of data collection. While this method may limit generalizability, the selected hospitals provided a relevant setting for investigating WPVs. A sample size of 50 nurses, representing approximately 25% of the total nursing workforce across these hospitals, was distributed as follows: Murzuq (*n* = 24), Tasawa (*n* = 12), and Aqar Otba (*n* = 14).

### Sampling

A convenience sampling method was employed, and nurses who were readily available and willing to participate in the study were selected. This approach was chosen because of its practicality and accessibility, given the time constraints and logistical challenges. A total of 50 nurses were included in the sample, representing those who were present during the data collection period and met the inclusion criteria.

### Data collection and study population

Data collection was conducted using a questionnaire adapted from a previously validated questionnaire [45]. The original validated questionnaire contained 37 items; however, for this study, we adapted 28 items and used them in our research. These included closed-ended questions, such as yes/no, and multiple-choice formats. The independent variables focused on demographic data, including gender and years of experience. The dependent variables explored workplace violence, covering topics such as the types and sources of violence, the impact of occupational violence on both professional and personal life, and strategies deemed effective for reducing workplace violence.

The study population comprised staff nurses of all ages who worked close to patients and were at high risk of exposure to workplace violence. Nurses working in the general surgical ward and surgical wards in the emergency department at Murzuq Hospital, as the other hospital (village hospital) does not have an emergency department, who were willing to participate were included, whereas those who were unwilling or unavailable during the study period were excluded. Data were collected over four weeks across three shifts via a self-administered questionnaire from the middle of May to June 2024.

### Data analysis

Descriptive statistics were employed to summarize the demographic and occupational characteristics of the nursing staff, presenting frequencies and percentages for categorical variables. Nonparametric inferential statistics were used to compare differences in experiences of occupational violence based on variables such as gender and years of experience. Chi-square tests were performed to examine associations between occupational violence and demographic or workplace factors. For subgroups with small sample sizes, Fisher’s exact test was used as an alternative. All the statistical tests were two-tailed, with the significance level set at *p* < 0.05. Data analysis was conducted via SPSS version 27.

### Ethical approval

Ethical approval for the study was obtained from the academic research committee of Fezzan University, with ID no. (FU/FN/24111), and official permission was granted by the hospital administration. Verbal and written informed consent were obtained from all participants before data collection. This study did not involve any experiments on humans or animals; however, it was conducted in accordance with the ethical principles outlined in the Declaration of Helsinki.

## Results

The demographic characteristics are presented in Table 1. The study included 50 nursing staff, with the majority being female (82%) and a smaller proportion being male (18%). In terms of age, 40% of the participants were between 30 and 39 years, followed by 26% in the 20–29 age range, 20% aged 40–49 years, and 14% aged 50 years or older. Most participants had over five years of experience (52%), whereas 28% had 3–5 years of experience, 12% had less than one year, and 8% had 1–2 years of experience. In the month before this study, 100% of the nursing staff reported that they had experienced some form of violence. Specifically, 80% experienced violence only once, 10% reported exposure several times a month, 8% experienced it once a week, and 2% were exposed daily. With respect to the types of violence encountered (Table 2), verbal insults were the most common, reported by 60% of the respondents, followed by discrimination (64%), threats (12%), and sexual abuse (2%). The primary sources of violence were the patient’s companion (68%), followed by the patients themselves (48%), coworkers (28%), administrators (12%), and doctors (2%) (Table 2). The impact of occupational violence on professional life was significant (Table 3), with 62% of participants reporting stress, 48% reporting a decrease in job satisfaction, 32% experiencing reduced performance, and 28% expressing a desire to leave their job. Similarly, the impacts on personal life included stress (62%), difficulty concentrating (40%), sleep disturbances (18%), and personal relationship problems (18%).

**Table 1:** Demographic data and exposure to violence (*n* = 50)

Item	Frequency	Percentage
**Gender**		
Male	9	18%
Female	41	82%
**Age**		
20–29 years	13	26%
30–39 years	20	40%
40–49 years	10	20%
50 years and more	7	14%
**Experience**		
Less than 1 year	6	12%
1–2 years	4	8%
3–5 years	14	28%
More than 5 years	26	52%
**Frequency of Exposure to Violence in the Past Month**		
Once	40	80%
Several times a month	5	10%
Once a week	4	8%
Daily	1	2%

**Table 2:** Types and sources of violence (*n* = 50)

Item	Frequency	Percentage
**Types of Violence**		
Verbal Insults	30	60%
Threats	6	12%
Sexual Abuse	1	2%
Discrimination	32	64%
**Sources of Violence**		
The Patient	20	48%
Patient’s Companion	34	68%
Coworker	14	28%
Doctor	1	2%
Administrator	6	12%

Multiple responses were possible

**Table 3:** Impact of occupational violence on professional and personal life (*n* = 50)

Item	Frequency	Percentage
**Impact on Professional Life**		
Feeling of Stress	31	62%
Decreased Satisfaction with Work	24	48%
Decrease in Performance	16	32%
Desire to Leave Work	14	28%
**Impact on Personal Life**		
Feeling of Stress	32	62%
Sleep Disturbances	9	18%
Difficulty Concentrating	20	40%
Problems in Personal Relationships	9	18%

Multiple responses were possible

Based on the opinions of nurses (Table 4), the factors contributing to workplace violence included a shortage of nurses (54%), aggressive behavior from patients or their companions (48%), equipment shortages (34%), and poor reception and treatment of patients (22%). The factors identified as contributing to professional violence were a lack of staff (40%), patient behavior (40%), work environment (28%), and inadequate training (32%), particularly in dealing with difficult situations (40%), and a lack of awareness of the importance of respecting nursing staff (62%). When asked about strategies to reduce occupational violence, 60% of the respondents emphasized the importance of training programs and psychological support, whereas 62% highlighted awareness campaigns. Additionally, 52% suggested improving the work environment and increasing the number of nurses. Statistical analysis revealed significant associations between gender and sources of violence (*p* ≤ 0.001), impact on professional life (*p* ≤ 0.001), and impact on personal life (*p* = 0.05). However, the association with the type of violence was not statistically significant (*p* = 0.06). These results are shown in Table 5. Similarly, (Table 6) experience was significantly associated with the type of violence encountered (*p* ≤ 0.001), the source of violence (*p* ≤ 0.001), and the impact of violence on both professionals (*p* ≤ 0.001) and personal life (*p* ≤ 0.001).

**Table 4:** Factors Contributing to Violence and Strategies to Reduce Violence based on the nurses’ viewpoints (*n* = 50)

Item	Frequency	Percentage
**Causes of Violence**		
Small Number of Nurses	27	54%
Shortage or Defect in Equipment	17	34%
Poor Reception and Treatment of Patients	11	22%
Aggressive Behavior from Patient/Attendant	24	48%
**Contributing Factors to Professional Violence**		
Workloads	6	12%
Lack of Staff	20	40%
Work Environment	14	28%
Patient Behavior	20	40%
Lack of Training	16	32%
Lack of Awareness about Respect for Nurses	31	62%
**Effective Strategies to Reduce Violence**		
Training Programs	30	60%
Policies to Prevent Violence	14	28%
Awareness Campaigns	31	62%
Improving Work Environment	26	52%
Increasing the Number of Nurses	26	52%
Providing Psychological Support	30	60%

Multiple responses were possible

**Table 5:** Association between Gender and Occupational Violence: Exploring Types, Sources, and Its Impact on Professional and Personal Life (*n* = 50)

Variable	Items	Gender		Total (%)	P value
		Male (%)	Female (%)		
Types of Violence	Verbal Insults	(3, 6%)	(27, 54%)	30 (60%)	0.06*
	Threats	(1, 2%)	(5, 10%)	6 (12%)	
	Sexual Abuse	(0, 0%)	(1, 2%)	1 (2%)	
	Discrimination	(5, 10%)	(27, 54%)	32 (64%)	
Sources of Violence	The Patient	(4, 8%)	(16, 32%)	20 (40%)	≤ 0.001*
	Patient’s Companion	(8, 16%)	(26, 52%)	34 (68%)	
	Coworker	(3, 6%)	(11, 22%)	14 (28%)	
	Doctor	(0, 0%)	(1, 2%)	1 (2%)	
	Administrator	(2, 4%)	(4, 8%)	6 (12%)	
Impact on Professional Life	Feeling of Stress	(9, 18%)	(22, 44%)	31 (62%)	≤ 0.001**
	Decreased Work Satisfaction	(6, 12%)	(18, 36%)	24 (48%)	
	Decrease in Performance	(4, 8%)	(12, 24%)	16 (32%)	
	Desire to Leave Work	(2, 4%)	(12, 24%)	14 (28%)	
Impact on Personal Life	Feeling of Stress	(9, 18%)	(23, 46%)	32 (62%)	0.05**
	Sleep Disturbances	(2, 4%)	(7, 14%)	9 (18%)	
	Difficulty Concentrating	(5, 10%)	(15, 30%)	20 (40%)	
	Problems in Personal Relationships	(2, 4%)	(7, 14%)	9 (18%)	

^*^ Fisher’s exact test was applied

** Chi-square test was applied

**Table 6:** Association between years of experience and occupational violence: Exploring types, sources, and their impact on professional and personal life (*n* = 50)

Variable	Items	Years of Experience				Total (%)	P value
		< 1 year	1–2 years	3–5 years	> 5 years		
Types of Violence	Verbal Insults	(3, 6%)	(2, 4%)	(8, 16%)	(17, 34%)	30 (60%)	≤ 0.001*
	Threats	(1, 2%)	(1, 2%)	(2, 4%)	(2, 4%)	6 (12%)	
	Sexual Abuse	(0, 0%)	(0, 0%)	(1, 2%)	(0, 0%)	1 (2%)	
	Discrimination	(3, 6%)	(3, 6%)	(9, 18%)	(17, 34%)	32 (64%)	
Sources of Violence	The Patient	(2, 4%)	(1, 2%)	(6, 12%)	(11, 22%)	20 (40%)	≤ 0.001*
	Patient’s Companion	(4, 8%)	(2, 4%)	(8, 16%)	(20, 40%)	34 (68%)	
	Coworker	(1, 2%)	(1, 2%)	(4, 8%)	(8, 16%)	14 (28%)	
	Doctor	(0, 0%)	(0, 0%)	(0, 0%)	(1, 2%)	1 (2%)	
	Administrator	(1, 2%)	(0, 0%)	(1, 2%)	(4, 8%)	6 (12%)	
Impact on Professional Life	Feeling of Stress	(3, 6%)	(2, 4%)	(9, 18%)	(17, 34%)	31 (62%)	≤ 0.001**
	Decreased Work Satisfaction	(2, 4%)	(1, 2%)	(6, 12%)	(15, 30%)	24 (48%)	
	Decrease in Performance	(1, 2%)	(1, 2%)	(4, 8%)	(10, 20%)	16 (32%)	
	Desire to Leave Work	(1, 2%)	(0, 0%)	(3, 6%)	(10, 20%)	14 (28%)	
Impact on Personal Life	Feeling of Stress	(3, 6%)	(2, 4%)	(8, 16%)	(19, 38%)	32 (62%)	≤ 0.001**
	Sleep Disturbances	(1, 2%)	(1, 2%)	(2, 4%)	(5, 10%)	9 (18%)	
	Difficulty Concentrating	(2, 4%)	(1, 2%)	(6, 12%)	(11, 22%)	20 (40%)	
	Problems in Personal Relationships	(1, 2%)	(0, 0%)	(3, 6%)	(5, 10%)	9 (18%)	

^*^ Fisher’s exact test was applied

** Chi-square test was applied

## Discussion

The findings of this study provide valuable insights into the prevalence and impact of occupational violence experienced by nursing staff in surgical wards. Key factors such as gender, experience, and workplace conditions play a significant role in shaping these experiences, with critical implications for both professional and personal outcomes. However, no statistically significant association was found between gender and the type of violence (*p* = 0.06), suggesting that male and female nurses may experience similar types of violence in this context. These results diverge from those of previous studies, such as one conducted in Nepal, where most victims of violence were males [46]. Different studies have reported varying experiences of workplace violence among male and female nurses, although the nature and impact of these experiences can differ significantly. For example, male nurses have been shown to report greater instances of violence from staff, whereas female nurses are more likely to face severe forms of harassment, such as sexual harassment. A study reported that, compared with 41% of male nurses, 67% of female nurses experienced sexual harassment [47]. Additionally, male nurses often report higher levels of burnout and lower job satisfaction, which are linked to workplace violence [48, 49]. Both genders encounter lateral violence, yet male nurses frequently lack organizational support in addressing these issues [49, 50]. While the type of violence may not significantly vary by gender, the consequences and reporting mechanisms differ, highlighting the need for tailored interventions in nursing environments.

Significant associations were observed between gender and the sources of violence (*p* ≤ 0.001) as well as the impact of violence on both professional life (*p* ≤ 0.001) and personal life (*p* = 0.05). This suggests that while the type of violence may not differ by gender, its sources and consequences do. Female nurses were more likely to report violence from patients’ companions and coworkers, a finding that is consistent with previous studies that highlight how gendered power dynamics exacerbate the exposure of female healthcare workers to violence from colleagues and patient family members [51]. Sociodemographic factors, the organizational climate, and workstation-specific risks have been identified as key contributors to WPV, particularly in emergency and surgical wards [10, 52]. Younger HCWs (under 40 years old), those in lower occupational positions, and those working night shifts face greater risks. Marital status also influences vulnerability: single or widowed nurses are more likely to experience harassment, whereas married nurses report higher instances of bullying [10, 51, 52]. WPV is particularly prevalent in emergency and surgical wards, with common perpetrators, including patient relatives, colleagues, and supervisors. The consequences for health are severe, ranging from psychological stress, depression, and anxiety to physical injuries such as fractures and lacerations [53, 54]. In patriarchal societies, such as Libya, traditional gender roles often position women as caregivers and men in authoritative roles, making female nurses more vulnerable to violence from patients or their families, who may hold differing expectations of respect and authority. This perception is heightened by the belief that female nurses have less authority than their male counterparts do, which can lead to aggression, especially when care expectations are not met [55–58].

The relationship between years of experience and workplace violence was highly significant (*p* ≤ 0.001), suggesting that more experienced nurses may have better coping mechanisms or that prolonged exposure to violent environments results in more significant personal and professional consequences over time. Female nurses with more than five years of experience constituted the majority of the study group, allowing for a comprehensive examination of how experience influences the occurrence and impact of violence. Discrimination was the most common form of violence reported, surpassing verbal insults. This aligns with findings from similar studies, which have noted that discrimination, often based on gender, age, or experience, can lead to feelings of exclusion and unfair treatment [53, 54]. Nurses with 6–10 years of experience have been reported to face greater exposure to lateral violence from coworkers, whereas those with less experience encounter different types of aggression [54]. In a cross-sectional study in Riyadh, 44.4% of nurses experienced some form of violence, with psychological abuse being the most prevalent [53]. A literature review highlighted a 110% increase in violent injuries among healthcare workers over the past decade, emphasizing the detrimental effects on mental health and job satisfaction [59].

The impact of occupational violence on professional life was substantial, with 62% of participants reporting stress due to violence, 48% reporting decreased job satisfaction, and 32% indicating a decline in work performance. Additionally, 28% expressed a desire to leave their job, reflecting the negative impact on workforce retention and stability. These findings align with broader literature linking WPV to burnout, decreased job satisfaction, and increased turnover intentions among healthcare workers [60]. Similarly, the psychological toll of WPV on personal life was profound. 62% of the respondents reported feeling stressed, 40% had trouble concentrating, and 18% reported sleep disturbances and problems in personal relationships. These results echo those of previous studies documenting the associations between WPV and psychological distress, anxiety, and depression [61, 62]. Exposure to violence is a known predictor of burnout, exacerbating symptoms of anxiety and depression and, in some cases, leading to PTSD [63–65]. From a professional standpoint, WPV negatively impacts job performance, leading to increased medical errors and decreased productivity, affecting patient care quality [66, 67]. On a personal level, the stress from WPV often spills over into healthcare workers’ personal lives, affecting relationships and overall well-being [64]. Victims of WPV may also experience physical symptoms, further complicating their health and personal lives [68].

The most common sources of violence were patients’ companions and patients themselves, which is consistent with other studies showing that patient families and companions are significant sources of violence due to misunderstandings or dissatisfaction with care [69–72]. Aggressive behavior from patients is often linked to stress, anxiety, and pain, which can lead to frustration and violent actions toward healthcare staff. Coworkers and administrators were also reported as sources of violence, highlighting the role of organizational dynamics in exacerbating WPVs. Physicians are less commonly reported as sources of violence, although power imbalances and conflicts over decision-making can still contribute to tension and aggression. Understanding the sources of WPV, including patient companions, patients, and coworkers, requires context-specific analysis to grasp how these groups contribute to violence in healthcare settings. While WPV is a well-documented issue in surgical wards, it is also prevalent in other high-risk healthcare settings, such as psychiatric wards, where the nature of care poses additional challenges. Workplace violence against nurses in psychiatric wards is a significant concern, with prevalence rates ranging from 40 to 90.6%, predominantly involving verbal (86.8%) and physical (57.5%) assaults [28, 73, 74]. This violence not only threatens nurses’ immediate safety but also has severe psychological consequences, including stress, emotional exhaustion, and avoidance behaviors [28, 75]. Several factors contribute to the high incidence of workplace violence, including peak times of aggression, poor organizational policies, and the nature of psychiatric care [76]. Additionally, drug abuse among patients is a significant trigger for violent incidents [28]. While the psychological toll on nurses is evident, some argue that workplace violence is an unavoidable aspect of psychiatric nursing, emphasizing the need for systemic changes rather than focusing solely on individual training [76]. To fully understand the complexities of WPV across different healthcare settings, it is crucial to examine the various sources of violence. In surgical wards, patient companions play a particularly significant role in escalating tensions.

Patient companions often escalate into verbal abuse or physical aggression due to dissatisfaction with treatment outcomes, long waiting times, or unmet expectations, particularly in high-stress environments such as surgical wards. Cultural norms emphasizing familial authority may also lead companions to feel entitled to control or influence the care process, contributing to violent incidents [55–58]. Patients may exhibit violent behavior due to factors such as pain, fear, mental health conditions, or substance abuse. In patriarchal societies, male patients may direct aggression toward female nurses as a display of dominance or frustration [10, 77–79]. Violence from coworkers, including hierarchical conflicts, bullying, or gender biases, is another critical source, often stemming from workplace dynamics. Female nurses may face harassment from senior staff or male colleagues, who perceive them as subordinates, a situation exacerbated by inadequate reporting mechanisms or fear of retaliation [10, 52].

Several factors contributing to WPV, including a lack of awareness of the importance of respecting nursing staff and understanding, were identified. These findings underscore the need for systemic changes in workplace policies, staffing levels, and training to address both the causes and effects of violence in healthcare settings. Research indicates that patient-related factors, such as high levels of pain, unmet expectations, and overcrowded hospitals, trigger aggressive behaviors from patients and their families [69–72]. Organizational issues, including poor policies, insufficient training, and staff shortages, contribute to the prevalence of WPV by exacerbating tensions [70–72]. Additionally, low sociocultural awareness among patients and their families can lead to misunderstandings and conflicts, further fuelling violent incidents [55, 56]. Stressful work environments, overcrowding, and long waiting times increase the risk of WPV for both patients and nurses, whereas communication breakdowns among healthcare staff add to misunderstandings and escalate conflicts [69–72]. Less experienced nurses are particularly vulnerable to WPV and lack the necessary skills to de-escalate potentially violent situations [69]. Some argue that WPV is an inevitable aspect of healthcare, emphasizing the need for resilience and coping strategies as essential tools to manage its impact [71].

In terms of prevention, the participants identified several strategies that they believed would be effective in reducing WPV, including awareness campaigns, training programs, and psychological support. Improving the work environment and increasing the number of nurses were also seen as critical measures. These findings align with the growing consensus in the literature that multifaceted interventions, including both organizational and individual-level strategies, are necessary to address the root causes of WPV and support healthcare workers [55, 72]. To mitigate WPV among nurses, a multifaceted approach focusing on education, communication, and organizational culture is crucial. Effective strategies include implementing educational programs to increase awareness of WPV and its consequences, as well as training nurses in communication skills to improve interactions with patients and their families, thus reducing misunderstandings that can lead to violence [56, 57, 80, 81]. Organizational policies, such as a zero-tolerance approach to violence and comprehensive reporting systems, are vital for promoting a culture of respect and ensuring that nurses feel supported when reporting incidents [71, 72, 82–84]. Environmental modifications, such as reducing hospital overcrowding and improving patient care protocols, can also help alleviate stressors contributing to WPV [56, 72]. However, systemic issues such as staffing shortages and high patient-to-nurse ratios may undermine the effectiveness of these strategies, requiring broader reforms in healthcare policy and management. Resilience-building helps nurses cope with WPV, reduces its psychological impact, and sustains quality care. Hospitals can foster resilience through peer support groups, counselling services, stress management workshops, and debriefing sessions after incidents [58, 77–79]. Leadership training for supervisors can build trust and encourage incident reporting. On a personal level, nurses can use mindfulness, stress reduction, assertive communication, and emotional intelligence to navigate high-stress situations [76, 85–89]. Professional development and mentorship further increase confidence and equip nurses with tools to handle aggression. While systemic changes may take time, resilience building provides immediate support and complements broader institutional efforts to create safer work environments.

### Limitations

This study has several limitations that may affect the generalizability of its findings. The small sample size (*n* = 50) limits statistical power and representativeness. Additionally, the use of convenience sampling may introduce selection bias, as participants were chosen based on availability rather than random selection, potentially overrepresenting certain characteristics and limiting diversity. The focus on surgical wards in a specific region may not reflect workplace violence dynamics in other departments or healthcare settings. The reliance on self-reported data also introduces potential biases, including underreporting due to fear of retaliation or memory lapses. Furthermore, the cross-sectional design prevents conclusions about causality or long-term effects of occupational violence.

## Conclusion

Occupational violence poses a significant challenge to nursing staff in surgical wards, as it affects personal well-being and professional efficacy. Gender, experience, and workplace factors significantly influence these experiences, necessitating targeted interventions. To address these issues, healthcare organizations should implement gender-sensitive policies, conduct training on conflict resolution, and provide psychological support. Future research should focus on longitudinal studies to explore the long-term effects of violence and assess the effectiveness of prevention strategies across diverse healthcare settings. Additionally, cultural and regional variations must be considered when tailoring interventions that address specific vulnerabilities and promote safer work environments.

## Data Availability

The datasets used and/or analyzed during the current study are available from the corresponding author upon reasonable request.
